# PrEP uptake and adherence in relation to HIV-1 incidence among Kenyan men who have sex with men

**DOI:** 10.1016/j.eclinm.2020.100541

**Published:** 2020-09-09

**Authors:** Elizabeth W. Wahome, Susan M. Graham, Alexander N. Thiong'o, Khamisi Mohamed, Tony Oduor, Evans Gichuru, John Mwambi, Prof. Maria Prins, Elise van der Elst, Prof. Eduard J. Sanders

**Affiliations:** a KEMRI/Wellcome Trust Research Programme Centre for Geographic Medicine Research–Coast, P.O. Box 230-80108, Kilifi, Kenya; b Departments of Global Health, Medicine, and Epidemiology, University of Washington, Seattle, Wash, USA; c Department of Infectious Diseases, Public Health Service of Amsterdam, Amsterdam, the Netherlands; d Department of Infectious Diseases, Amsterdam Infection & Immunity Institute (AI&II), Amsterdam UMC, University of Amsterdam, Amsterdam, the Netherlands; e Department of Global Health, University of Amsterdam, Amsterdam, the Netherlands; f Nuffield Department of Medicine, University of Oxford, Headington, UK

**Keywords:** MSM, Pre-exposure prophylaxis, Tenofovir, HIV-1 incidence, Kenya

## Abstract

**Background:**

Data on HIV-1 incidence following programmatic pre-exposure prophylaxis (PrEP) uptake by men who have sex with men (MSM) are limited in sub-Saharan Africa.

**Methods:**

Since June 2017, MSM participating in an ongoing cohort study in Kenya were offered daily PrEP, assessed for PrEP uptake and adherence, and evaluated for HIV-1 acquisition monthly. We determined tenofovir-diphosphate (TFV-DP) concentrations in dried blood spots 6–12 months after PrEP initiation, and tenofovir (TFV) concentrations and genotypic drug resistance in plasma samples when HIV-1 infection occurred. We assessed HIV-1 incidence by reported PrEP use.

**Findings:**

Of 172 MSM, 170 (98·8%) were eligible for PrEP, 140 (82·4%) started it, and 64 (57·7%) reported PrEP use at end of study. Of nine MSM who acquired HIV-1 [incidence rate: 3·9 (95% confidence interval (CI), 2·0–7·4) per 100 person-years (PY)], five reported PrEP use at the time of HIV-1 acquisition [incidence rate: 3·6 (95% CI, 1·5–8·6) per 100 PY)] and four had stopped or had never started PrEP [incidence rate: 4·3 (95% CI, 1·6–11·3) per 100 PY]. Among 76 MSM who reported PrEP use, 11 (14·5%) had protective TFV-DP concentrations of ≥700 fmol/punch (≥4 tablets a week). Among the five MSM who acquired HIV-1 while reporting PrEP use, only one had detectable but low TFV concentrations in plasma and none had genotypic HIV-1 resistance.

**Interpretation:**

HIV-1 incidence among MSM with access to programmatic PrEP was high and did not differ by reported PrEP use. Only one in seven MSM taking PrEP had protective tenofovir concentrations and four out of five MSM who acquired HIV-1 while reporting PrEP use had not taken it. Strengthened PrEP adherence support is required among MSM in Kenya.

**Funding:**

This work was supported by the International AIDS Vaccine Initiative (IAVI).

**Research in context**

**Evidence before this study**

We searched PubMed between 01/01/1999 to 31/03/2020 for English language articles on PrEP, uptake, HIV, incidence, men who have sex with men, Africa using the search terms “HIV”, “incidence”, “Africa”, “pre-exposure prophylaxis uptake”, “PrEP”, “adherence”, “men who have sex with men”, “MSM”. We found several studies on HIV incidence among MSM in Africa, but these studies were not done in the context of PrEP for HIV prevention. Although PrEP has been recommended for use in prevention of HIV acquisition and has been made available in a few countries in Africa, our search did not identify any evaluations of PrEP uptake and adherence in relation to HIV acquisition among MSM in Africa.

**Added value of this study**

Our results show that while PrEP uptake among men who have sex with men (MSM) was high, the HIV-1 incidence rate was substantial (3•9 per 100 PY) and did not differ between men reporting PrEP use and those who had never started PrEP or discontinued it, suggesting that PrEP adherence was poor. We found that among participants taking PrEP for at least 6 months only one in seven had sufficient PrEP taken to achieve protection against HIV-1 infection, and that four out of five MSM who acquired HIV-1 while reporting PrEP use had not taken it.

**Implications of all the available evidence**

We have established that MSM in Kenya face challenges in daily PrEP taking, and alternative strategies to support PrEP adherence, including long-acting PrEP or on-demand PrEP strategies are urgently needed for MSM.

Alt-text: Unlabelled box

## 1 Introduction

Kenya has the fifth largest number of people living with HIV-1 in the world [1], with an estimated 1·3 million people infected with HIV-1 [2]. In 2018, there were 36,000 new HIV-1 diagnoses [2], of whom nearly 40% were young adults between 15 and 24 years [3]. Timely linkage to care, increased antiretroviral therapy (ART) coverage and scale-up of interventions to improve health outcomes among individuals living with HIV-1 has led to a significant decline in the number of new infections [4]. However, key populations (KP) including men who have sex with men (MSM), female sex workers (FSW), and people who inject drugs continue to be severely impacted by HIV-1 [5]. Since May 2017, the Kenya Ministry of Health (MoH), through the National AIDS and STI Control Program (NASCOP) has been rolling out pre-exposure prophylaxis (PrEP) nationally to individuals at ongoing risk of HIV-1 acquisition [6]. Counties with an estimated high or medium HIV-1 incidence rate in the general population and those with a high or medium number of KP (including MSM) are prioritized for PrEP roll-out [7].

Mathematical models using local epidemiological and behavioral data provide insights on which approach would yield the greatest reduction of HIV-1 transmission [8], [9], [10]. Results from a modelling study using data from Nairobi suggested that, in a setting with limited resources, decreasing overall HIV-1 infections could best be achieved when male sex workers were prioritized for PrEP and ART initiation, followed by prioritizing MSM not reporting sex work and FSW [10]. While Kenyan guidelines engage KP for PrEP initiation and sexually transmitted infections (STI) prevention [11], there is little information available on whether male sex workers or MSM will actually take up PrEP when offered, and continue using it as prescribed.

Prior to PrEP availability in Kenya, the HIV-1 incidence estimated among adult male sex workers in Nairobi was 10·9 per 100 person-years (PY) [12]; among MSM in coastal Kenya, of whom a substantial proportion (70%) reported sex work, incidence was estimated at 7·0 per 100 PY [13]. While hypothetical PrEP interest was very high (98%) amongst MSM and transgender women (TGW) in a recent study in coastal Kenya [14], only one clinical trial has been conducted in MSM in Kenya [15], [16], [17]. In this study, conducted in Nairobi and coastal Kenya in 2012, high PrEP acceptance was reported among at-risk MSM [16], and median adherence to daily PrEP was higher than median adherence to intermittent PrEP use (83% vs. 55%, *P*<0·003) [17]. This clinical trial also assessed qualitative barriers to PrEP adherence among at-risk MSM, including stigma, side effects, pill size, and individual lifestyle factors such as frequent travelling [16].

Data on PrEP uptake and adherence in relation to HIV-1 incidence are needed to improve programmatic PrEP implementation in Kenya. The objectives of the present study were to estimate PrEP uptake, adherence, continuation, and HIV-1 incidence among MSM who were offered PrEP programmatically in coastal Kenya.

## 2 Methods

### 2.1 Study design and participants

This study was conducted using data from an HIV-1 vaccine preparedness open cohort study described previously [18]. The study was set-up in a research clinic located in Mtwapa town about 20 km north of Mombasa, the second largest town in Kenya. Mtwapa is known for its busy nightlife characterized by hotels, nightclubs and bars, and an active 24-hour economy [19]. The town has an estimated population of approximately 90,677 [18,20]. Participants were recruited by 10–15 trained peer mobilizers through their personal networks and at venues where sex workers meet to establish contact with clients [18]. Adults aged 18–49 years were eligible if they met any of the following criteria: HIV-1-negative and reporting any of transactional sex work, a recent STI, multiple sexual partners, sex with an HIV-1-infected partner, or anal sex during the 3 months before enrolment [18]. This analysis includes all follow-up visits for men reporting same-sex behavior between June 19, 2017 and June 30, 2019. All participants provided written informed consent. The KEMRI Ethics Review Committee approved the study.

### 2.2 Procedures

Cohort enrollment and follow-up visits included a standardized face-to-face interview using a risk behavior questionnaire, HIV-1 testing and counseling using rapid point of care antibody tests, risk-reduction counselling, medical history, and physical examination [18]. Participants were offered hepatitis B vaccination and syndromic treatment for STIs at baseline and follow-up, following Kenyan Ministry of Health guidelines [21].

### 2.3 PrEP assessment

Since June 19, 2017, daily PrEP (tenofovir-disoproxil-fumarate combined with emtricitabine) has been offered to eligible MSM cohort participants at follow-up visits [22]. HIV-1 negative participants were assessed for PrEP eligibility by either a cohort-derived HIV-1 risk score (age 18–24 years, having only male sex partners, any receptive anal intercourse (RAI), any unprotected sex (defined as insertive or receptive anal sex, or vaginal sex) in the past week and group sex) [13], or MoH criteria (a sexual partner who is HIV-1 positive or has unknown HIV-1 status, transactional sex, a recent STI, recurrent use of post exposure prophylaxis, having sex while under the influence of alcohol, inconsistent condom use, and sharing needles among people who inject drugs) [6]. Participants eligible for PrEP were counselled about benefits and risks of PrEP and the importance of adherence, and educated about recognizing symptoms of acute HIV infection. Those who were willing to start PrEP were provided with a 30-day PrEP supply. During monthly visits, participants taking PrEP completed a questionnaire on PrEP knowledge, motivation to take PrEP, and PrEP adherence via audio computer-assisted self-interview (ACASI) in either English or Swahili. Refills were provided at each monthly visit to all participants continuing PrEP. At participant visits between July 2018 and November 2018, a one-time dried blood spot (DBS) sample was collected for assessment of tenofovir-diphosphate (TFV-DP) and emtricitabine-triphosphate (FTC-TP) concentration. For participants who acquired HIV-1 while taking PrEP, a plasma sample already collected at the time of HIV-1 diagnosis was assessed for tenofovir (TFV) concentration.

### 2.4 Laboratory evaluation

At each visit, two rapid antibody test kits (Determine: Abbott Laboratories, Abbott Park, Illinois, USA; Unigold; Trinity Biotech plc, Bray, Ireland) were used in parallel for HIV-1 testing. Discordant rapid HIV-1 test results were resolved using HIV-1 RNA (Xpert® HIV-1 Qual, Cepheid). Starting in 2016, if symptoms of acute HIV (fever, fatigue, diarrhea, body pains, sore throat, or genital ulcers) or risk criteria (any RAI, group sex, or age (18–29 years)) were met, Xpert® HIV-1 RNA Qual, Cepheid testing was performed.

Gonococcal infection was diagnosed among participants who reported urethral or rectal symptoms by the detection of Gram-negative, intracellular diplococci consistent with *Neisseria gonorrhoeae* in urethral or rectal secretions [18]. Prevalent syphilis infection was diagnosed by a positive rapid plasma reagin (RPR, tested annually) titre confirmed by Treponema pallidum haemagglutination assay (TPHA). Incident syphilis was defined as a four-fold increase in RPR titre confirmed by TPHA.

DBS were stored at −20 °C within 24 h of collection and later shipped on dry ice to the Anderson laboratory in Denver, Colorado, USA. TFV-DP and FTC-TP were quantified from a 3-mm punch using a previously validated methodology with an assay range from 25 to 6000 fmol/sample for TFV-DP and 0·1 to 200 pmol/sample for FTC-TP [23].

Plasma samples collected at the time of HIV-1 diagnosis from participants who acquired HIV-1 while on PrEP were sent to Clinical Laboratory Services in Johannesburg, South Africa. Quantification of TVF in plasma was performed by mass spectrometry and the lower limit of quantification (LLOQ) was set at the concentration of the lowest validated standard for tenofovir, 10·0 ng/mL.

Genotypic drug resistance mutations among participants who acquired HIV-1 while on PrEP were identified from partial HIV-1 *pol* sequences containing the protease and part of the reverse transcriptase genes. Sequences were interpreted using the Stanford HIV-1 drug resistance database and the WHO list for surveillance of drug resistance strains [24].

### 2.5 Measures

#### 2.5.1 Definitions

PrEP uptake was defined as acceptance of PrEP and PrEP initiation by an eligible participant. PrEP baseline was defined as the first study visit by a participant during June 19, 2017 – June 30, 2019. At each visit, participants who received a PrEP refill at their previous visit and wanted to continue PrEP during their current visit or those who received a PrEP refill at their previous visit, but discontinued PrEP due to an HIV-1 positive test result on their current visit were categorized as PrEP users. Those not receiving a PrEP refill in their previous visit and those who were discontinuing, re-starting, or starting PrEP during their current visit were categorized as not taking PrEP at that visit. Participants were defined as lost to follow-up (LTFU) if they were late by >90 days for their scheduled appointment date.

#### 2.5.2 Socio-demographic and behavior characteristics

Data collected at enrollment included previously identified predictors of HIV-1 acquisition in this cohort, including age (18–24 or 25+ years), sexual behavior in the past week (no sexual activity, all protected, any unprotected sex), sex of sexual partner through-out the study (men having sex with men exclusively or men having sex with both men and women), and any RAI, group sex, or receiving payment for sex with cash, living expenses, or goods in the past 3 months (yes/no) [13].

#### 2.5.3 Concentration of TFV-DP and FTC-TP in DBS and TFV in plasma

The concentrations of TFV-DP and FTC-TP in DBS were evaluated as a continuous measure in fmol per punch. Dosing categories for TFV-DP were defined as: below the limit of quantification (BLQ), LLOQ to 350 fmol/punch (<2 tablets per week), 350 to 699 fmol/punch (2 to 3 tablets per week), 700 to 1250 fmol/punch (4 to 6 tablets per week), and >1250 fmol/punch (7 tablets a week). Protective concentrations of TFV-DP were defined as ≥700 fmol/punch consistent with taking ≥4 tablets a week [25]. Concentration of FTC-TP in DBS was defined as BLQ or quantifiable (consistent with a recent PrEP dosing within 36–48 h) [26]. The concentration of TFV in plasma for participants who acquired HIV-1 while reporting PrEP use was assessed as a continuous measure and categorized as: BLQ, 10 ng/mL (LLOQ), >10 ng/mL (consistent with dosing in the last 2–3 days), and >40 ng/mL (consistent with dosing within 24 h) [27].

### 2.6 Statistical analysis

Descriptive statistics were used to summarize baseline demographic and behavioral characteristics of MSM enrolled in the study. Data for each participant were censored at their last monthly follow-up visit before the censoring date (June 30, 2019) or, for those who acquired HIV-1 infection during follow-up, the estimated date of infection calculated as: 10 days before the sample collection date when the sample tested positive for HIV-1 RNA and negative for HIV-1 serology or the mid-point between a previously negative and subsequently positive HIV-1 serologic test when the sample with HIV-1 negative serology tested negative for HIV-1 RNA or when HIV-1 RNA testing was not performed [18]. Participants who were ineligible for PrEP at baseline but became eligible during follow-up (*N* = 3) were included in the analysis and only observations following the occurrence of eligibility were included in the analysis. Total observation time was obtained by adding up separate observation times for all participants in the study between June 19, 2017 and June 30, 2019 and expressed in terms of PY. HIV-1 incidence rates were calculated as the number of incident HIV-1 cases divided by PY of follow-up and expressed as incidence per 100 PY. *P* values were 2-sided, and statistical significance was set at *P*<0·05. Data were cleaned, recoded, and analyzed using Stata 15·0 (StataCorp LLC, College Station, Texas, USA).

### 2.7 Role of the funding source

The funders of the study had no role in study design, data collection, data analysis, data interpretation, or writing of the report. The corresponding author had full access to all the data in the study and had final responsibility for the decision to submit for publication.

### 2.8 STROBE guidelines

This article adheres to the STROBE guidelines.

## 3 Results

Between June 2017 and June 2019, out of 179 HIV-1 negative registered MSM cohort participants, seven (3·9%) MSM made no follow up visits and two (1·1%) were not eligible for PrEP throughout the study period and were excluded. Of the 170 HIV-1 negative MSM eligible for PrEP and who made at least two study visits, the median age was 25 years (range, 19–44), most (150 [88·2%]) were never married and 42 (24·7%) reported having sex with men exclusively during the follow-up period. At baseline, nearly three-quarters (127 [74·7%]) reported any RAI in the past 3 months, over half (95 [55·9%]) reported <100% condom use for any anal sex in the past three months, and nearly two-thirds (111 [65·3%]) received payment for sex in the past 3 months (Table 1).

**Table 1:** Baseline characteristics of 170 HIV-1-negative MSM, Kilifi, Kenya, June 2017-June 2019.

Characteristics	n (%)
Age (years), median (range)	25 (19–44)
Age group (years)	
18–24	70 (41.2)
25+	100 (58.8)
Education	
Primary/none	66 (38.8)
Secondary	89 (52.4)
Higher/tertiary	15 (8.8)
Marital status	
Never married	150 (88.2)
Ever married	20 (11.8)
Religion	
Christian	86 (50.6)
Muslim	45 (26.5)
Other/none	39 (22.9)
Employment	
None	29 (17.1)
Self	115 (67.6)
Formal	26 (15.3)
Sex of sexual partners*	
Men and women	128 (75.3)
Men exclusively	42 (24.7)
Sexual behavior, past week	
No activity	67 (39.4)
100% condom use	69 (40.6)
<100% condom use	34 (20.0)
Sexual activity past week†	
Number of sexual partners [Median (IQR)]	1 (0–2)
Number of sexual acts [Median (IQR)]	2 (0–4)
Receptive anal intercourse (RAI), past 3 months	127 (74.7)
Insertive anal intercourse (IAI) past, 3 months	116 (68.2)
Condom use for any anal sex, past 3 months	
No activity	6 (3.5)
100% condom use	69 (40.6)
<100% condom use	95 (55.9)
Paid for sex with cash, living expenses, or goods, past 3 months	28 (16.5)
Received payment for sex with cash, living expenses, or goods, past 3 months	111 (65.3)
Group sex, past 3 months	3 (1.8)
Alcohol use, past month	74 (43.5)
Sex after alcohol use, past month	50 (29.4)
Recent sexually transmitted infection‡	1 (0.6)

Note. These analyses include participants who were eligible for PrEP and contributed at least two follow-up visits. IQR=interquartile range.

⁎ Assessed throughout the follow-up period.

† Among those reporting having sex.

‡ Defined as detection of Gram-negative, intracellular diplococci in urethral or rectal secretions or rectal secretions or a new syphilis diagnosis within 6 months.

### 3.1 PrEP uptake, PrEP continuation, and LTFU

**Fig. 1:**
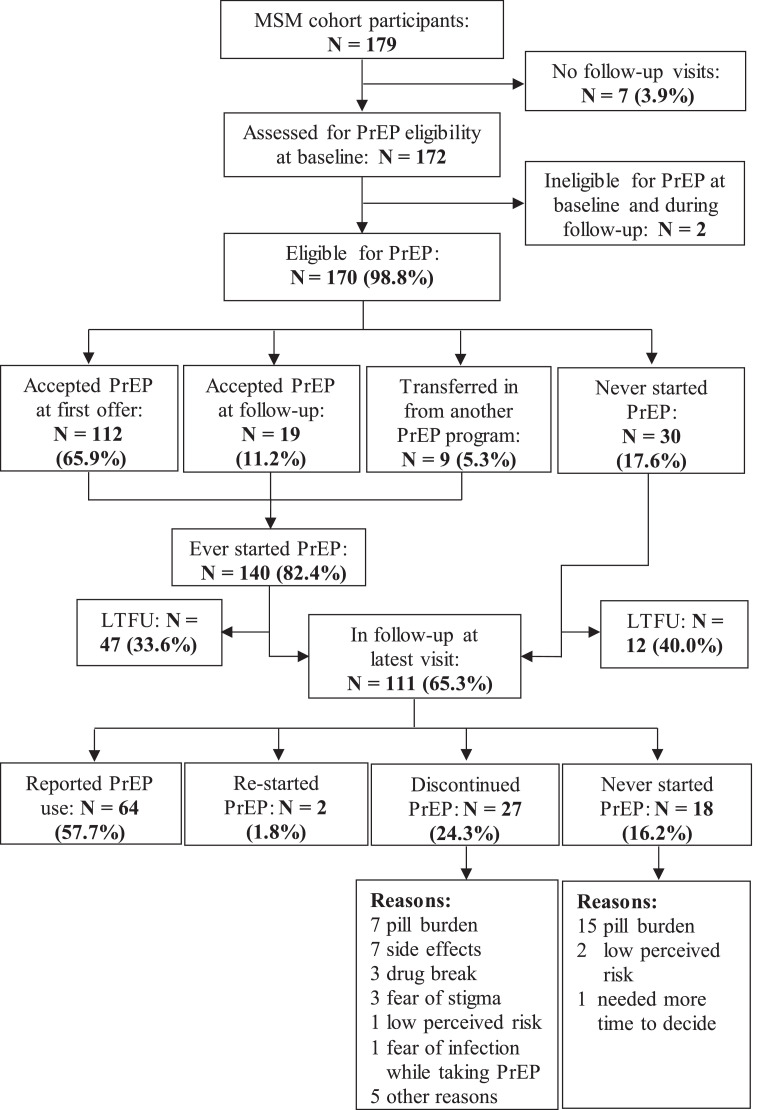
**Flow of PrEP uptake and reported PrEP use among 179 MSM, Kilifi, Kenya, June 2017-June 2019.** A total of 140 participants ever started PrEP including 9 who transferred in from another PrEP-providing facility. The median follow-up time for 59 participants who were LTFU was 7.3 months, interquartile range 2•5–13•8 months). PrEP=pre-exposure prophylaxis; LTFU=loss to follow-up.

Of the 172 MSM assessed for PrEP eligibility, 170 (98·8%, 95% confidence interval (CI), 95·9–99·9) were eligible for PrEP. One hundred and twelve (65·9%, 95% CI, 58·2–73·0) accepted at first offer and nine MSM transferred in from another PrEP-providing facility. Out of 49 MSM who did not accept PrEP at baseline, 19 (11·4%, 95% CI, 7·0–17·2) MSM accepted PrEP during follow-up visits, within a median of 83 (IQR 44–220) days. Overall, 111 (65·3%) eligible MSM were in follow-up at the censor date, at which point 64 (57·7%, 95% CI, 47·9–67·0) reported ongoing PrEP use and 47 (42·3%, 95% CI, 33·0–52·1) were not taking PrEP (including 27 who discontinued, two who were re-starting it and 18 who never started PrEP). The proportion LTFU among eligible MSM who ever started PrEP compared to that of eligible MSM who never started PrEP did not differ (33·6% vs 40·0%; *P* = 0·50) (Fig. 1).

### 3.2 HIV-1 incidence

**Fig. 2:**
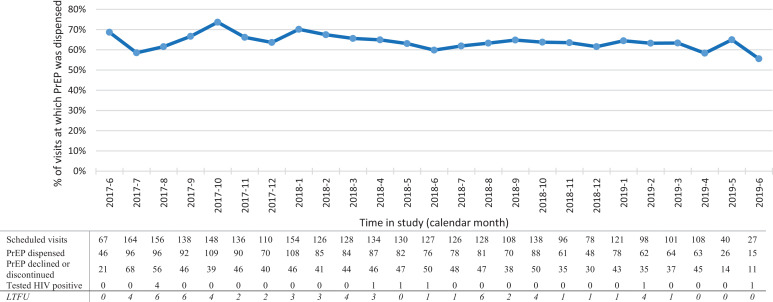
**Frequency of PrEP dispensing by month of study visit among 179 MSM, Kilifi, Kenya, June 2017-June 2019.** The figure illustrates the proportion of participants’ scheduled study visits at which PrEP was dispensed during follow-up. The table details the overall number of participants’ scheduled visits per calendar month, and the number of visits at which PrEP was dispensed. *LTFU* row shows the number of participants and the month at which they were LTFU. The number LTFU is not part of the scheduled visits. PrEP=pre-exposure prophylaxis, LTFU=loss to follow-up.

Overall, 170 MSM contributed 233·7 PY (median 21·2 months, IQR 9·5–22·2 months). Participants made a median of 21 study scheduled visits (IQR, 9–24 visits) and 18 scheduled visits (IQR, 7–22 visits) at which PrEP was dispensed. Overall, participants made a total of 2877 scheduled follow-up visits, of which 1859 (64·4%) occurred among participants who were prescribed PrEP, (Fig. 2).

**Fig. 3:**
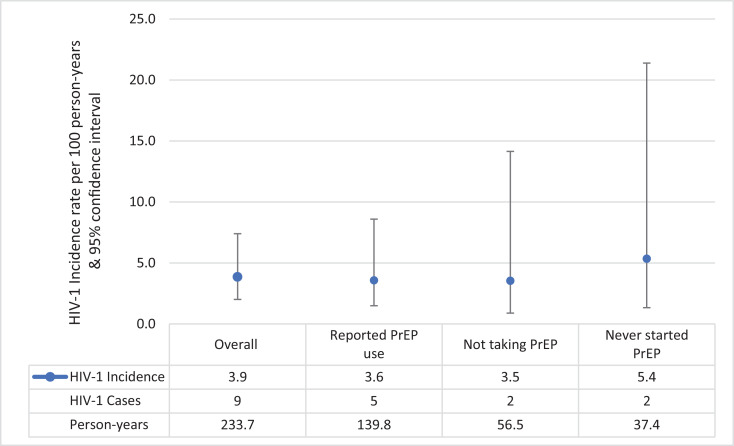
**HIV-1 incidence by reported PrEP use among 170 MSM, Kilifi, Kenya, June 2017- June 2019.** PrEP=pre-exposure prophylaxis.

A total of nine MSM acquired HIV-1 during follow-up, for an incidence rate of 3·9 (95% CI, 2·0–7·4) per 100 PY. Among MSM who ever started PrEP, seven acquired HIV-1, for an incidence rate of 3·6 (95% CI, 1·7–7·5) per 100 PY, including five who reported ongoing PrEP use, and two who had discontinued PrEP before their HIV diagnosis. Among MSM who never started PrEP, two acquired HIV-1, for an incidence rate of 5.4 (95% CI, 1·3–21·4) per 100 PY (Fig. 3).

### 3.3 PrEP adherence

TFV-DP and FTC-TP concentrations in DBS were measured for 76 MSM at a single visit between July 2018 and November 2018, corresponding to 6 and 12 months following PrEP initiation. Concentrations of TFV-DP and FTC-TP were detectable in samples from 32 (42·1%, 95% CI, 30·9–54·0) and 13 (17·1%, 95% CI, 9·4–27·5) of MSM, respectively. Out of 11 (14·5%, 95% CI, 7·4–24·4) MSM with TFV-DP concentrations of ≥700 fmol/punch, consistent with taking ≥4 tablets a week, eight (72·7%) had detectable FTC-TP concentrations, consistent with a recent (within 36–48 h) PrEP dosing. Of the 44 (57·9%, 95% CI, 46·0–69·1) MSM with undetectable TFV-DP concentrations, none had detectable FTC-TP concentrations (table 2). Of note, unprotected sex in the past week was reported by more participants with TFV-DP concentrations of ≥700 fmol/punch, compared to participants with TFV-DP concentrations of <700 fmol/punch (72·7%, 8/11 vs. 26·2%, 17/44, *P* = 0·02).

**Table 2:** Concentration of FTC-TP and TDF-DP in DBS among 76 MSM, Kilifi, Kenya, June 2017-June 2019.

	TDF-DP (fmol/punch)					
FTC-TP (fmol/punch)	BLQ (%)	LLOQ-349 (%)	350–699 (%)	700–1249 (%)	≥1250 (%)	Total (col%)
BLQ	44 (100.0)	14 (100.0)	2 (28.6)	2 (28.6)	1 (25.0)	63 (82.9)
Detectable	0 (0.0)	0 (0.0)	5 (71.4)	5 (71.4)	3 (75.0)	13 (17.1)
Total (row%)	44 (57.9)	14 (18.4)	7 (9.2)	7 (9.2)	4 (5.3)	76

TFV-DP=tenofovir-diphosphate; FTC-TP=emtricitabine-triphosphate; BLQ=below the limit of quantification; LLOQ=lower limit of quantification.

**Fig. 4:**
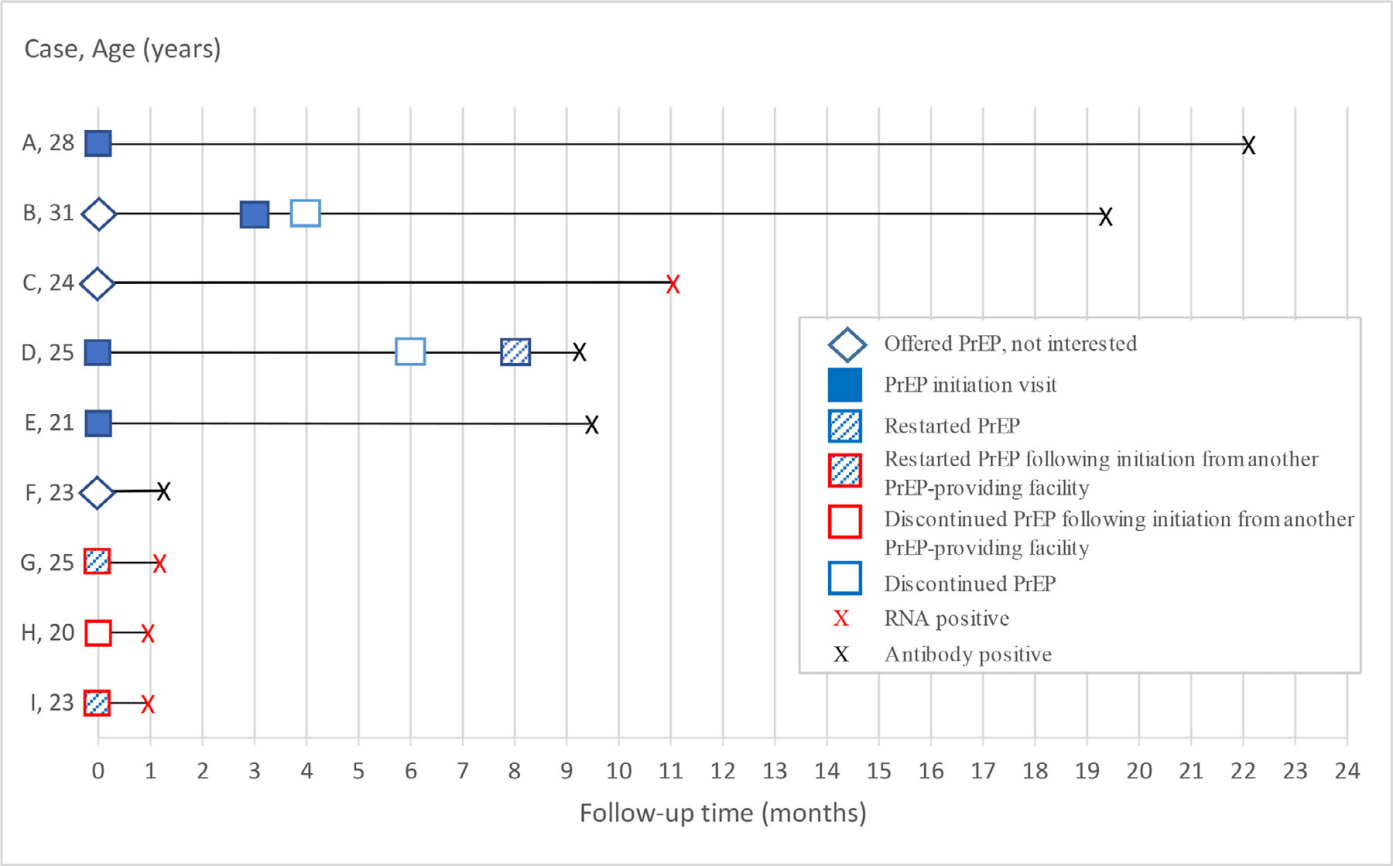
**Summary of HIV-1 acquisitions that occurred among nine participants during follow-up, Kilifi, Kenya, June 2017-June 2019. A** reported inconsistent PrEP use before HIV-1 infection visit, had undetectable TFV concentrations at HIV-1 infection visit. **B** was not interested in PrEP at first offer due to fear of side effects. Discontinued PrEP one month after initiation due side effects. **C** was not interested in PrEP due to pill burden. **D** discontinued PrEP after travelling away from study area, restarted 2 months later. Reported good adherence and had detectable but low TFV concentrations (66•0 ng/Ml) in plasma at HIV-1 infection visit. **E** reported good adherence but had undetectable TFV concentrations at HIV-1 infection visit. **F** was not interested in PrEP, needed more time to decide. **G** experienced side effects following PrEP initiation from another PrEP-providing facility, restarted PrEP following further counselling at study clinic, had undetectable TFV concentrations at HIV-1 infection visit. **H** discontinued PrEP due to side effects one month following initiation from another PrEP-providing facility. Was willing to restart at study clinic but tested HIV-1 positive. **I** discontinued PrEP due to poor adherence two months following initiation from another PrEP-providing facility. Restarted at study clinic but reported inconsistent PrEP use due to travelling and other life challenges. Had undetectable TFV concentrations at HIV-1 infection visit. PrEP=pre-exposure prophylaxis; TFV=tenofovir.

Among the five MSM (two RNA positive and three antibody positive) who acquired HIV-1 while reporting PrEP use, only one had detectable but low TFV concentrations in plasma (66·0 ng/mL, consistent with dosing in the last 1–2 days), and none had genotypic HIV-1 resistance (Fig. 4).

Of these five participants, three reported to have discontinued PrEP due to side effects, travel and other life challenges and restarted it on the visit before the HIV-1 infection visit (participants D, G, and I in Fig. 4).

## 4 Discussion

In this study, we document a substantial overall HIV-1 incidence rate (3·9 per 100 PY) among MSM with access to programmatic PrEP who were followed in a HIV-1 vaccine feasibility cohort study in coastal Kenya. While initial PrEP uptake was high, HIV-1 incidence did not differ between men reporting PrEP use and those who had never started PrEP or discontinued, suggesting that PrEP adherence was poor. Analysis of drug concentrations supported this conclusion: only four in ten MSM who took PrEP for at least 6 months had any PrEP detected. Only one in seven MSM was taking sufficient PrEP to achieve tenofovir concentrations consistent with taking ≥4 PrEP pills per week, and four out of five MSM who acquired HIV-1 while reporting PrEP use had undetectable TFV concentrations.

In this study, PrEP uptake within the first 3 months was high (81%). This high uptake may have been mainly attributed to increased PrEP awareness facilitated by the individual and group educational sessions conducted before PrEP became available to cohort participants [22]. Additionally, PrEP awareness has been found to be associated with PrEP acceptance globally [28]. While we did not assess reasons for willingness to use PrEP in this study, varying reasons have been cited among other MSM populations in sub-Saharan African (SSA) including; a sense of relief due to PrEP effectiveness, additional protection against HIV-1 infection, and motivation to remain HIV-1 negative and protect partner from HIV-1 infection [14,29].

In our setting, participants received monthly counselling on PrEP initiation or continuation and collected 30-day PrEP pills at monthly visits when interested to continue or to restart PrEP. This visit schedule is different from programmatic PrEP provision which offers 3-monthly supplies. Why some participants were collecting PrEP monthly and not taking it is not clear. It is possible that participants felt stigmatized if they were seen taking PrEP at home [30]. Participants may also have felt expectations from clinic staff to take PrEP. Qualitative research to assess reasons for not taking PrEP among MSM receiving PrEP is ongoing. During follow-up, about one in three MSM who started PrEP discontinued it, including two MSM who acquired HIV-1 in our study. Daily pill taking, stigma associated with taking PrEP, side effects, and low perceived risk were some of the reasons given for discontinuing PrEP, consistent with findings in our previous study and recent findings in a similar setting [14,16]. An analysis of predictors of switching on and off PrEP in this cohort is ongoing. While PrEP is not taken for life but during seasons of risk, individuals may stop and restart more than once over a refill period [31]. However, participants stopping PrEP may require guidance in switching to an alternative HIV-1 prevention strategy [32]. It should also be assessed whether Kenyan MSM would be more adherent to on-demand PrEP, as high levels of protection have been found in recent on-demand trials [33].

Estimates of TDF-DP concentrations in combination with FTC-TP concentrations in our study presents stark evidence that the majority of participants were collecting PrEP but not taking it at all [26]. Compared to participants who took some PrEP but had insufficient levels for protection, those few with protective PrEP levels reported statistically significantly more condomless sex, suggesting that they may have been motivated by their higher risk. Other than this potentially promising finding, our results are consistent with findings from a recent study conducted in a different community in coastal Kenya among 34 MSM and eight TGW, in which only three (8%) of participants had TFV-DP concentrations in blood plasma corresponding with ≥4 tablets a week [34]. In well-resourced settings, results from an open-label extension study among MSM and TGW enrolled at a municipal STI clinic in the US found high PrEP protective levels (80% - 82%) at follow-up visits over a similar duration. These high PrEP levels were mainly attributed to favorable community perception and a wide awareness of PrEP effectiveness [35]. In Kenya, where same-sex behavior is stigmatized, PrEP-taking individuals may benefit from a peer support model, which recently led to improved ART adherence among MSM in a pilot study in coastal Kenya [36]. In addition, interventions such as eHealth technologies and intensified engagement are needed to support retention of MSM in PrEP care which may improve PrEP adherence over time [37].

In this study, a high proportion of MSM who acquired HIV-1 while taking PrEP had undetectable tenofovir in blood plasma at the HIV-1 infection visit. A review of their study documents found that 3 MSM had re-started PrEP before the HIV-1 infection visit, suggesting that these men may have used PrEP intermittently during period of high risk. Of note, all nine HIV-1 acquisitions (including five who reported ongoing PrEP use and four who had discontinued or never started PrEP) occurred among MSM who reported any RAI. We have previously recommended that RAI should be added as an inclusion criterion for PrEP eligibility in Kenyan guidelines [22], to improve targeting and risk reduction counseling. Peer-delivered PrEP counseling could potentially reduce stigma and lead to improved confidence in sexual health among MSM, supporting informed decision-making on HIV-1 prevention strategies for use during periods of HIV-1 risk [31].

Our study had several limitations. Firstly, retention on PrEP was assessed through clinic-provided refills, which may not reflect PrEP adherence. However, we also used an objective PrEP adherence measure based on TFV-DP and FTC-TP concentrations in DBS collected at a single timepoint from MSM taking PrEP for at least 6 months. Secondly, while TFV-DP concentrations may quantify the average number of PrEP pills taken in the past week, this measure does not specify the pattern of PrEP taking during risky periods. We did not use a timeline follow-back or other recall measures to assess co-incidence between pill taking and condomless anal sex. Thirdly, PrEP users were counselled on using combination prevention strategies that involve both PrEP and condoms for prevention of bacterial STIs and HIV-1. It is possible that men used condoms instead of PrEP for HIV prevention. Fourthly, MSM followed up in this study reported high-risk sexual behavior, therefore the observed HIV-1 incidence may not be representative of all Kenyan MSM. Lastly, risk behavior may be affected by social desirability bias, and was collected during face-to-face interviews.

Our study has documented a substantial HIV-1 incidence among at-risk MSM with access to programmatic PrEP in coastal Kenya. Despite high PrEP uptake, participants who collected PrEP at monthly visits had low drug concentrations when assessed in DBS, suggesting MSM in Kenya face challenges in daily PrEP taking. Further research with involvement of the MSM community is needed to understand their perspective of PrEP. Alternative strategies to support PrEP adherence among MSM, including long-acting PrEP or on-demand PrEP strategies for MSM are urgently needed.

## Declaration of Competing Interest

Dr Prins reports grants from Gilead sciences, Roche, AbbVie, and MSD during the conduct of the study. All other authors have nothing to report.

## Appendix Supplementary materials

Image, application 1
